# Unusually high room and elevated-temperature tensile properties observed in direct aged wire-arc directed energy deposited Inconel 718

**DOI:** 10.1038/s41598-023-46674-z

**Published:** 2023-11-06

**Authors:** Jie Song, Xavier A. Jimenez, Carissa Russell, Albert C. To, Yao Fu

**Affiliations:** 1 Department of Aerospace and Ocean Engineering, Virginia Tech, Blacksburg, USA; 2 Department of Mechanical and Materials Engineering, University of Pittsburgh, Pittsburgh, USA; 3 Materials Sciences LLC, Horsham, USA; 4 Department of Materials Science and Engineering, Virginia Tech, Blacksburg, USA

**Keywords:** Engineering, Materials science

## Abstract

Wire-arc directed energy deposition (DED) processed Inconel (IN) 718 is known to have coarse columnar grains, strong texture, and significant chemical and microstructural inhomogeneity in the as-fabricated condition. Homogenization treatment is commonly used prior to aging to eliminate the inhomogeneity and detrimental precipitation for better mechanical properties. In this study, however, direct aging (DA) at 700 °C without homogenization has resulted in room-temperature yield strength, ultimate tensile strength (UTS), and elongation that are comparable to wrought condition and among the highest reported properties for wire-arc DED IN718. The DA samples at between 650 and 750 °C aging also demonstrates remarkable ductility when deformed at elevated temperatures. In addition, when aged below 750 °C the DA IN718 possesses significantly higher UTS compared to those with homogenization treatment. These superior mechanical properties are highly likely due to the non-uniform and hierarchical precipitation consisting of disk-shaped γ″ in diameter from a few to tens of nm in the dendritic core area and micron-sized Laves phase and carbides in the inter-dendritic region.

## Introduction

Inconel 718 (IN718) constituting 70% of the total Ni-based superalloys have been widely used in the hot sections of aerospace turbine engines, oil- and gas-field applications, and nuclear reactors^1^. This alloy demonstrates an excellent combination of high temperature mechanical and chemical stability, with a wide service temperature from cryogenic ~ − 250 °C to ~ 700 °C^2, 3^. IN718 derives its outstanding mechanical attributes mainly through the metastable γ″-Ni_3_(Nb, Ti) phase coherency strain hardening^4–6^. The γ′ phase of lower volume fraction also minorly contributes to its strengthening through chemical or order hardening^4–6^. IN718 exhibits superior anti-oxidation property at a wide range of temperature from 650 to 850 °C, through the formation of passive and continuous chromia at the outer surface^7–10^.

As IN718 is expensive and hard to machine due to its high strain hardening response, there has been great interest to develop a cost-effective free-forming manufacturing process to reduce materials wastage and machine cost. In the past decade, interest has grown remarkably in developing additive manufacturing (AM) techniques such as laser and electron beam powder bed fusion, and laser metal deposition^11–13^. The main disadvantage of the metal powder-based AM techniques is the low deposition rate (0.12–0.6 kg/h). As a high deposition rate and near net-shape manufacturing technique, wire-arc directed energy deposited (DED), that employs adapted welding equipment, requires low capital investment, and can achieve up to a hundred-time higher deposition rate. Therefore, it has recently evolved into a topic of immediate interest in the scientific and research community^14–17^.

There have been several studies to investigate the applicability of wire-arc DED to fabricate IN718. It has been found that a highly textured columnar grains of dendritic morphology along the buildup direction are formed by all different types of arc-welding techniques^18–26^. The solidification and cooling process need to be carefully controlled to remove the detrimental phases such as Laves and delta phase^27–29^. The tensile strength of wire-arc DED 718 has been found to be characteristically lower compared to the wrought alloy, attributed to the presence of Laves phase and extensive δ phase precipitation^19,30^. Interpass rolling, that applies cold rolling and introduces plastic deformation to the top deposit during the layer-by-layer deposition, has been adopted to improve the mechanical properties under plasma process-based wire arc, and appeared to be effective in transforming most of the columnar grain structure into equiaxed grains and increasing the yield strength^22^. In order to eliminate the detrimental laves phase, post-deposition solution heated treatment has been explored as a beneficial approach to eliminate Laves phase by dissolving it back into the γ-matrix^30^. A high homogenization temperature, however, promotes excessive grain growth and can lower the mechanical strength^30^.

Overall, there is still a lack of understanding in the relation between microstructure and high temperature tensile behaviors of wire-arc DED 718. Moreover, the heat treatment effect on the microstructures and mechanical properties requires better understanding to optimize the post-processing procedures. In this work, different aging temperatures are applied to the as-fabricated (AF) 718 and compared with that first treated with homogenization process. Their microstructures and mechanical properties at room and elevated temperatures are investigated.

## Results

**Figure 1:**
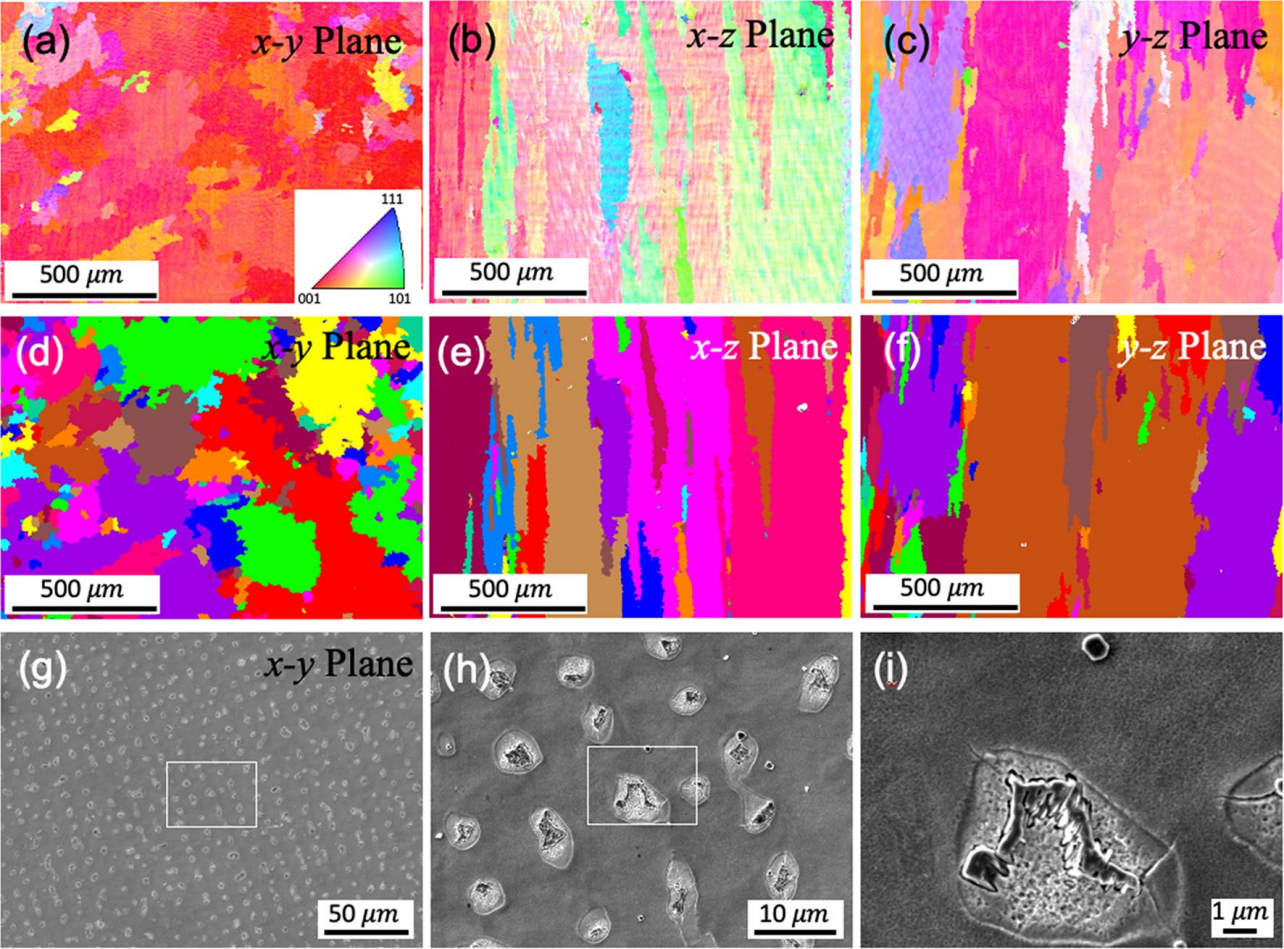
As-fabricated (AF) conditions: (**a**–**c**) EBSD-IPF images demonstrate the grain orientation and (**d**–**f**) EBSD-grain images demonstrate the grain structures on the different build planes. (**g**–**i**) SEM images show the inter-dendritic region and precipitates; the image of (**h**) and (**i**) are the magnification of the white rectangle domain in (**g**) and (**h**), respectively.

**Figure 2:**
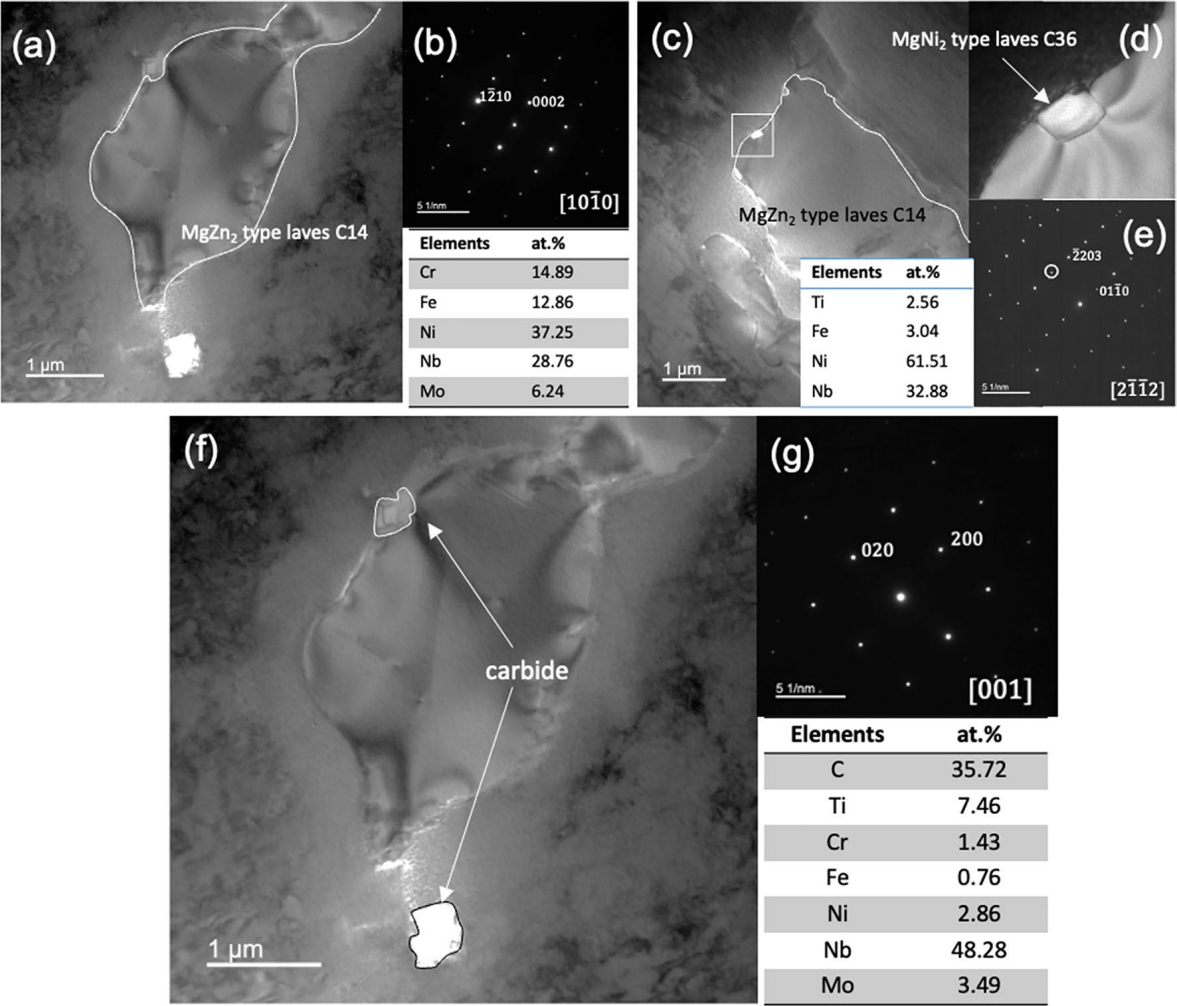
As-fabricated conditions: (**a**–**e**) TEM images, SAD pattern and EDS analysis of the Laves phases; the contours of the Laves phase have been highlighted in white line. (**f**, **g**) TEM images demonstrate the inter-dendritic Nb-rich carbide precipitates, SAD pattern, and EDS analysis; the contour of the carbide is highlighted in white/black line.

The chemical composition of the wire-arc DED 718 determined by energy dispersive X-ray spectroscopy (EDS) is 53.6Ni–21.3Cr–18.8Fe–3.1Nb–1.8Mo–2.4Ti (at%), falling into the typical range for IN718. The AF IN718 develops a typical feature of epitaxial growth in the wire-arc DED material with large γ columnar grains of up to several millimeters along the build direction (BD) (Fig. 1a–f). A strong texture on the *x–y* plane is developed with preferential {100} crystallographic growth direction in the largest thermal gradient direction (approximately along the build direction). The non-uniform distribution of solute element across the solid–liquid interface, due to the low partition coefficient (0.25–0.5) of Nb in IN718 from 700 to 1000 °C^31^, leads to the inter-dendritic area enriched with Nb. The formation of the Laves phases and Nb-rich carbides is facilitated by the element segregation in the inter-dendritic area (Fig. 1g–i). The presence of irregularly shaped Laves phase and submicron-carbides has been confirmed by the TEM selected area electron diffraction (SAD) pattern. Two types of Laves phases of different lattice structures and chemical compositions are identified as shown in Fig. 2a–e. The irregularly shaped Laves phases of type C14 are larger in size and of MgZn_2_ structure. The sub-micron Laves phase of type C36 is of MgNi_2_ type and has significantly reduced Cr and Fe and higher Ni element. The Ni-rich carbides shown in TEM images also contains high Ti element as revealed by the EDS analysis (Fig. 2f,g).

**Figure 3:**
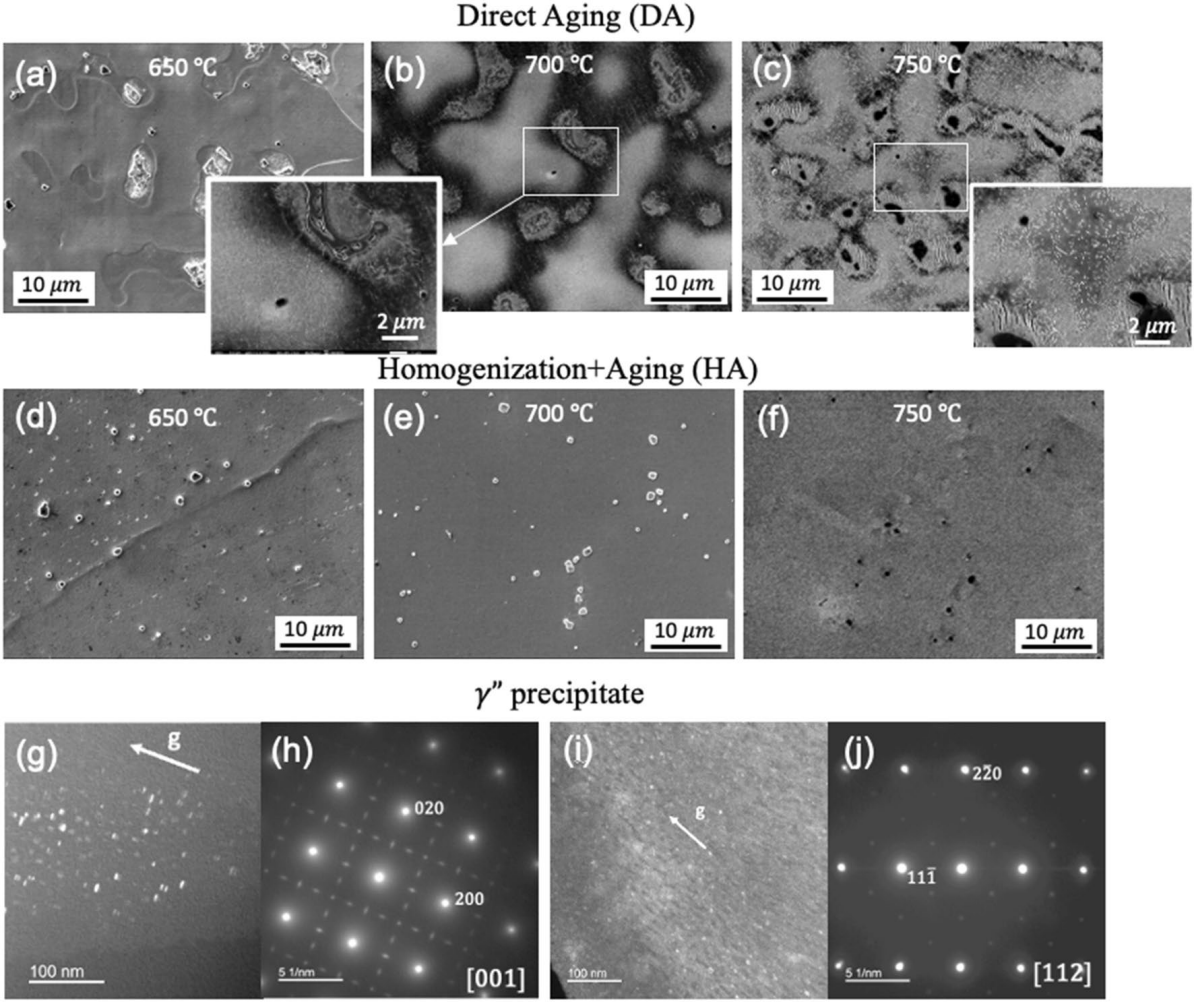
SEM images of the DA 718 at the (**a**) 650 °C, (**b**) 700 °C, and (**c**) 750 °C aging; and SEM images of the HA 718 at the (**d**) 650 °C, (**e**) 700 °C, and (**f**) 750° aging; insert is the magnified image of the white rectangular domain. (**g**–**j**) TEM images and SAD patterns of the nanosized γ″ precipitates in the 700 °C aged samples. Aging time is 16 h (16H).

Direct aging (DA) at below 650 °C leads to no noticeable change in the microstructures (Fig. 3a). The details of DA process are described in the Method section. The γ″ on the order of a few nanometer forms at the 600 °C and 700 °C aging. The γ″ precipitates are still too small to be identified by SEM at this stage. The light-colored inter-dendritic area in Fig. 3b is likely caused by different chemical etching behaviors during SEM sample preparation, and thus contrast in microstructures between the dendritic core and inter-dendritic area (Fig. 3b). Using TEM, the γ″ precipitates can be observed as small discs of diameter ~ 10 nm and thickness ~ 2 nm (Fig. 3g–j). At 750 °C (Fig. 3c) and 800 °C (Fig. S2a in supplementary document), the sub-micron sized γ″ unevenly precipitates in the γ matrix, with higher density in the dendrite core area. In addition, δ phases start to appear close to the inter-dendritic area surrounding the Laves phase and carbides (Fig. 3c and Fig. S2a in supplementary document).

In the homogenization + aging (HA) samples, most of Nb-rich inter-dendritic phase dissolves into the matrix, accompanied with the disappeared dendrite morphology and more uniform chemical composition. The details of HA process are described in the Method section. The grain size is comparable to that for the AF condition (Fig. S1 in supplementary document). Cuboidal carbides do not dissolve into the matrix (Fig. 3d–f). The main types of precipitation (γ″, δ phase) in the HA condition at the different aging temperatures coincide with that in the DA condition. However, the precipitates distribution is much more uniform (Fig. 3f), attributed to the chemical and microstructural homogenization process. The aspect ratio of the δ phase is significantly larger compared to that in the AF conditions at above 800 °C (Fig. S2b,d in supplementary document). The phase constitution and their distribution in response to aging is similar between the wrought + HA (HAw) and HA conditions. The main difference is the grain boundary discontinuous Cr_23_C_6_ carbide precipitate present in the HAw condition (Fig. S2e–g in supplementary document) whereas the carbides are in cuboidal shape in the HA samples (Fig. 3d–f). It is also noticeable that the precipitates are slightly more refined in the HAw condition at below 800 °C, where the γ″ of nanometer size at the 750 °C aging cannot be identified in the SEM (Fig. S2e in supplementary document).

**Figure 4:**
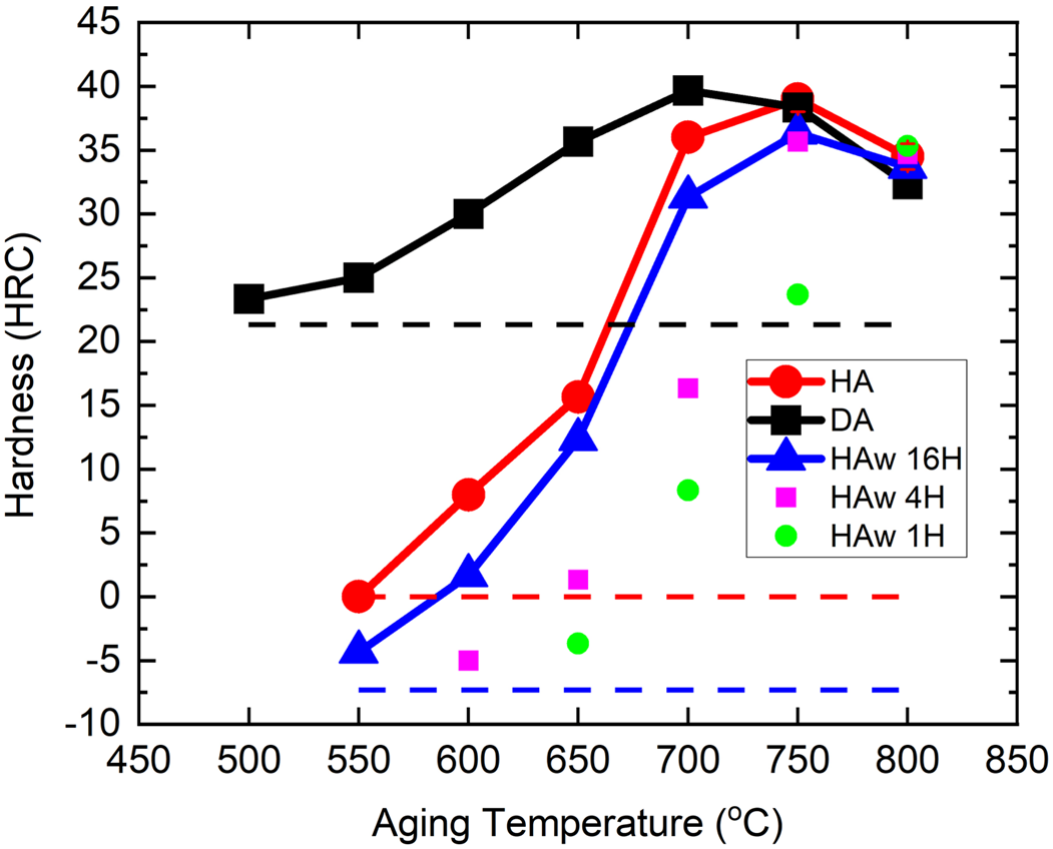
Hardness as a variation of aging temperature in the DA, HA, and HAw 718 samples; dashed lines are the hardness of the AF samples before aging at the corresponding condition. Aging time is 16 h (16H) unless marked differently in the figure.

The hardness of DA, HA, and HAw conditions as a function of the aging temperature is shown in Fig. 4, with their corresponding unaged conditions displayed in the dashed lines as reference. Note the negative HRC values are only provided here for comparison purpose. At the HAw condition, the hardness variation with time has been investigated after 1, 4, and 16 h aging. A gradual increase in hardness is observed due to the relatively sluggish γ″ precipitation. A 16-h aging is thus employed for comparison among the fabricated conditions to ensure a sufficient time for γ″ precipitation without significant coarsening. When not heat-treated and aged below 700 °C, DA IN718 has a higher hardness compared to that of the HA and HAw ones, attributed to the inter-dendritic laves phase (Ni, Cr, Fe)_2_(Nb, Mo, Ti), Ni_2_Nb, and Nb-rich Nb(Ti)C. The hardness in all the conditions demonstrates an increase until 700–750 °C followed by a decrease at a higher aging temperature. The increased hardness is associated with the nanometer-sized γ″ precipitates whereas the decreased hardness is likely associated with their coarsening as well as the nucleation and growth of δ precipitates. Aging at and below 700 °C leads to a higher hardness in the AF condition, whereas the hardness in samples aged between 750 and 800 °C is comparable among different conditions.

**Figure 5:**
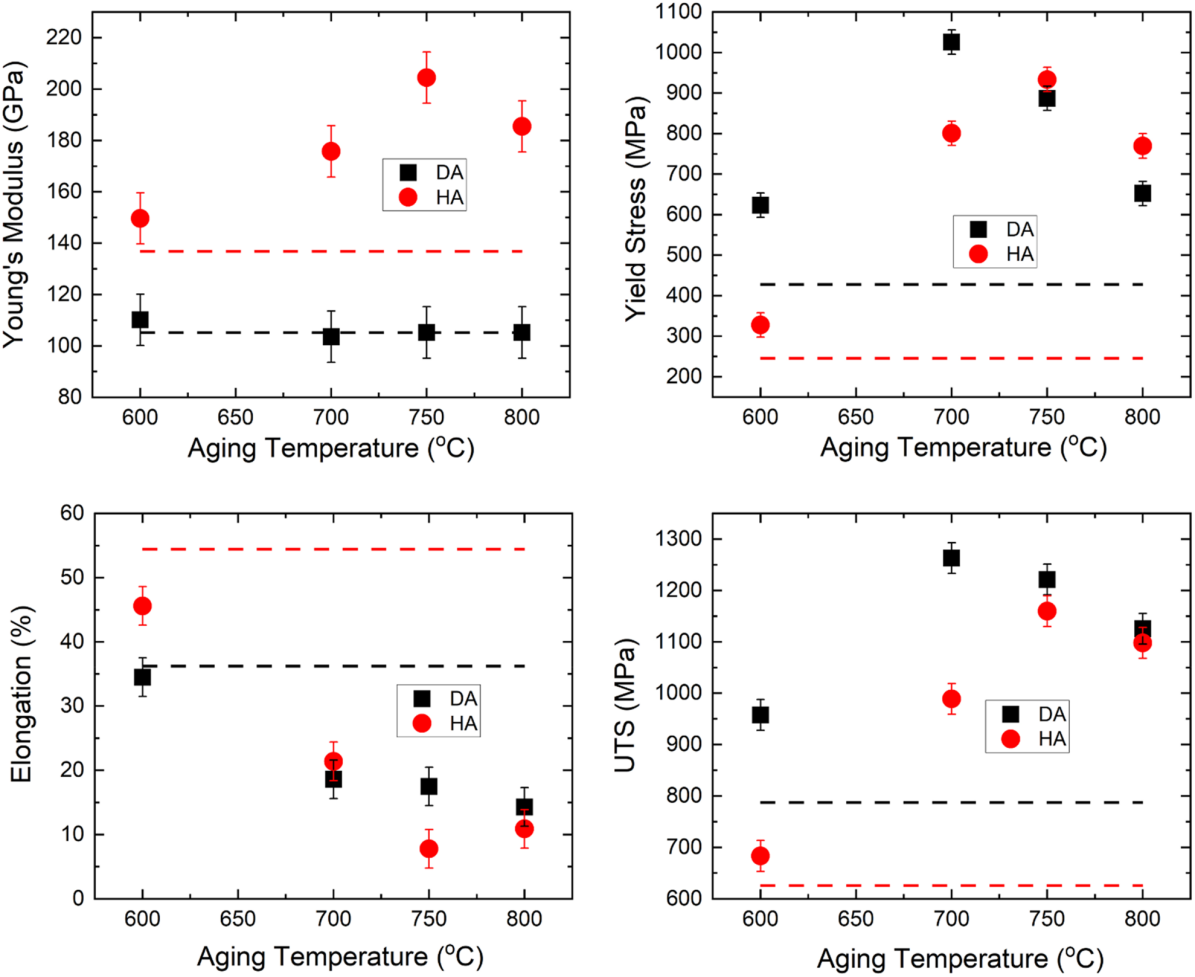
Mechanical properties including Young’s modulus, yield strength (YS_0.2_), elongation, and ultimate yield strength (UTS), extracted from the room temperature tensile loading test; dashed lines are the corresponding properties before aging. Aging time is 16 h unless marked differently in the figure.

**Figure 6:**
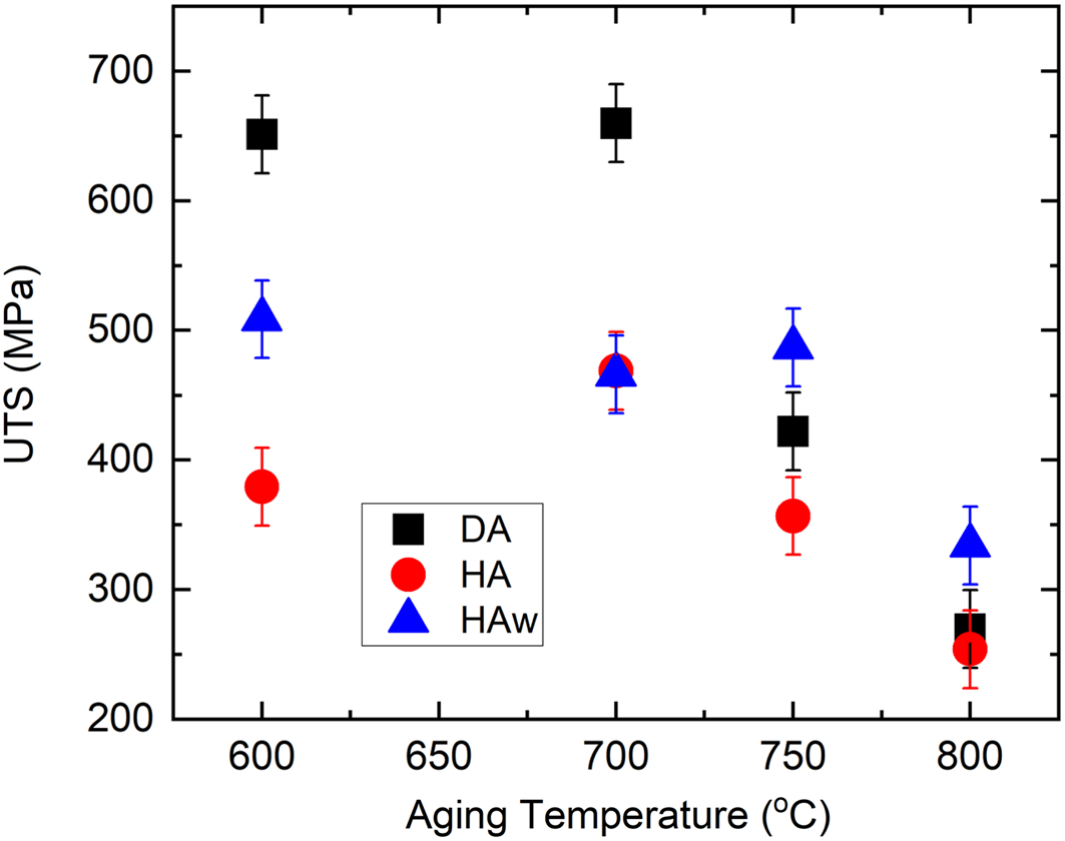
Ultimate tensile strength extracted from the elevated temperature tensile loading test; tensile testing temperature is the same as the aging temperature. Aging time is 16 h unless marked differently in the figure.

The mechanical properties extracted from the room temperature tensile tests are summarized in Fig. 5. The dashed line are the properties prior to aging. The AF samples possess a room-temperature Young’s modulus of 105.2 GPa, 0.2% offset yield strength (YS_0.2_) of 427.7 MPa, ultimate tensile strength (UTS) of 787.4 MPa, and a high ductility of 36.2%, comparable to those reported in the previous studies^22,32,33^. The Young’s modulus in the DA condition is considerably lower than that of the HA conditions, and does not vary with the different aging temperatures. In contrast, the Young’s modulus of the HA condition is increased to 136.9 GPa and responds to the aging treatment, reaching over 200 GPa after aging at 750 °C. The YS_0.2_ and UTS of the DA sample at the 600–750 °C aging are higher or comparable to the HA ones at room temperature. The elongation at fracture in general decreases with increasing aging temperatures. The lowest ductility of lower than 10% is found in the HA 718 at a 750 °C aging. The DA 700 °C has reached a room-temperature YS_0.2_ of 1029.0 MPa, UTS of 1263.1 MPa and a ductility of 18.6%, comparable to the wrought standard^34^. The tensile behaviors at elevated temperatures are also investigated and the extracted UTS are shown in Fig. 6. It should be noted that the tensile loading is conducted at the same temperature as the aging temperatures so no significant change in microstructures is expected during the high temperature tensile tests. When aged between 600  and 700 °C, the AF IN718 possesses a UTS higher than the conditions with homogenization (HA and HAw). The UTS at 750 °C and above with different processing conditions are comparable to each other.

## Discussion

**Figure 7:**
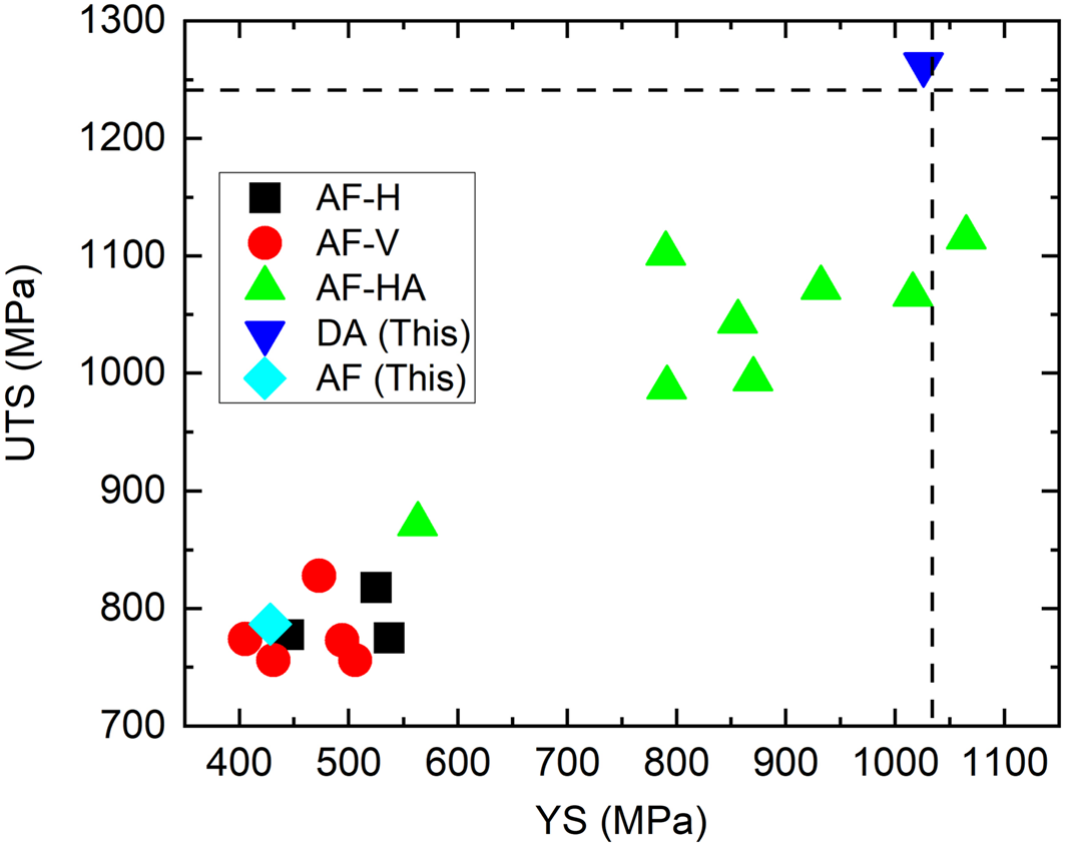
Comparison of the room temperature YS_0.2_ and UTS of our study with data reported in literature and equivalent conventional materials^19,22,23,30,32,33^. ‘AF’ represents as-fabricated condition; ‘HA’ represents as-fabricated followed by heat treatment with aging; ‘This’ represents the current study and the aging is at 700 °C; ‘AF-H’ and ‘AF-V’ represent the tensile sample in the horizontal (x–y) plane and vertical (z) direction, respectively. The wrought standard condition^34^ is represented by the dashed line.

It is apparent that the DA at 700 °C leads to noticeably higher mechanical properties (YS_0.2_ and UTS) at room temperature, compared to the HA and HAw ones at the same aging and tensile conditions. A comparison of the mechanical properties with reported values of wire-arc DED 718 at room temperature in the literature is made in Fig. 7. Even though the AF condition has a comparable YS_0.2_ and UTS of slightly lower values than those in other studies, the properties after direct aging at 700 °C are significantly improved and among the highest values reported so far (Fig. 7). Seow has also reported a high YS (1065 MPa) and UTS (1116 MPa) achieved by standard heat treatment to wire-arc DED 718^30^. However, that condition suffers from an extremely low ductility of 0.9%^30^, whereas the DA at 700 °C in our study maintains a good ductility of 18.6%. This finding is in contrast to the previously reported inferior mechanical properties of wire-arc DED 718 compared to the homogenization treated and wrought ones, often attributed to the presence of Laves and δ phases^22,32,33^. In our study, the microstructural characterization of the AF sample clearly shows the presence of the Laves phase and highly non-uniform microstructural features (Fig. 3b,c), whereas the Laves phase has largely dissolved into the matrix of the HA samples which possess a more uniform chemical and microstructural features (Fig. 3e,f). Even though grains coarsen during homogenization treatment and grow to twice as large, the grain boundary strengthening effect is overall insignificant considering the large columnar grain after fabrication, and less likely to be responsible for the difference in mechanical properties.

**Figure 8:**
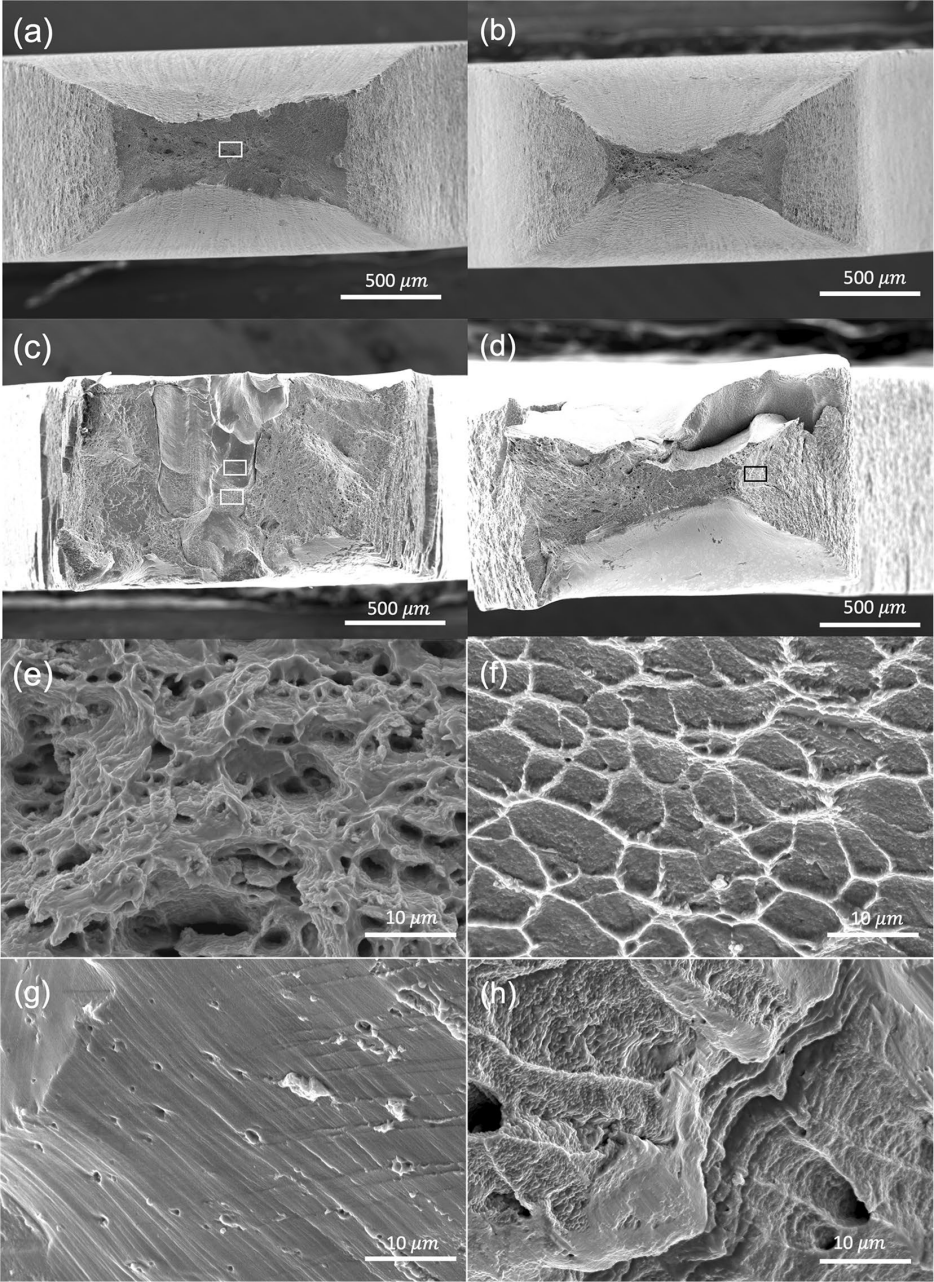
Fractography of DA samples after (**a**) 700 °C, (**b**) 750 °C tensile test; (**e**) magnified image of rectangular area in (**a**); HA samples after (**c**) 700 °C, (**d**) 750 °C tensile test. (**f**–**h**) magnified image of rectangular area in (**c**) and (**d**). Note that the aging temperature is the same as the tensile test temperatures.

At the high temperature tensile test, the DA sample demonstrates a higher UTS than the HA and HAw ones after aging at the temperature below 750 °C. In addition, a superior ductility at the elevated temperature has been observed in the DA samples as shown in Fig. 8. The cross-section area reduces remarkably to approximately 8–10% at 700 °C and 3–5% at 750 °C of the original cross section area (Fig. 8a,b). It should be noted that the UTS is calculated based on the original cross-section area, and the true stress is significantly higher due to the drastic plastic deformation. The fractography shows largely homogeneous features of continuous plastic flow followed by dimples ruptures through micro-void coalescence (Fig. 8a,b,d). The fractured surface in the HA samples, instead, shows more complex mixed features with cleavage facets, river markings and small domains of ductile fracture (Fig. 8c,d,f–g). The corresponding area reduces to approximately 30–40% at 700 °C and to 13–18% at 750 °C. The microstructural features should be responsible for the observed differences in mechanical properties. At elevated temperatures, it is apparent that the Laves phase and dendritic feature do not lead to the early fracture and reduced ductility in the DA condition. Rather, the hierarchical and patterned precipitates (nanoscale γ″ precipitates and microscale Laves and carbides) appear to contribute to the superior ductility and increased strength. The grain structure and grain boundaries are more likely to play a less significant role due to the large grain size of 0.5–1 mm in the AF and the DA condition. EBSD analysis also revealed an equiaxed grain structure with a comparable grain size (~ 0.5 mm) after the homogenization treatment (Fig. S1 in the supplementary document). Since the main source of strength comes from precipitation and the grain boundary strengthening is rather limited, it is less likely that the grain structure is responsible for the exceptional mechanical properties. Future studies will be needed to further clarify the deformation mechanism and specific roles of multiple precipitation phases at the room and high temperature deformation.

## Methods

### Wire-arc DED deposition procedure

**Figure 9:**
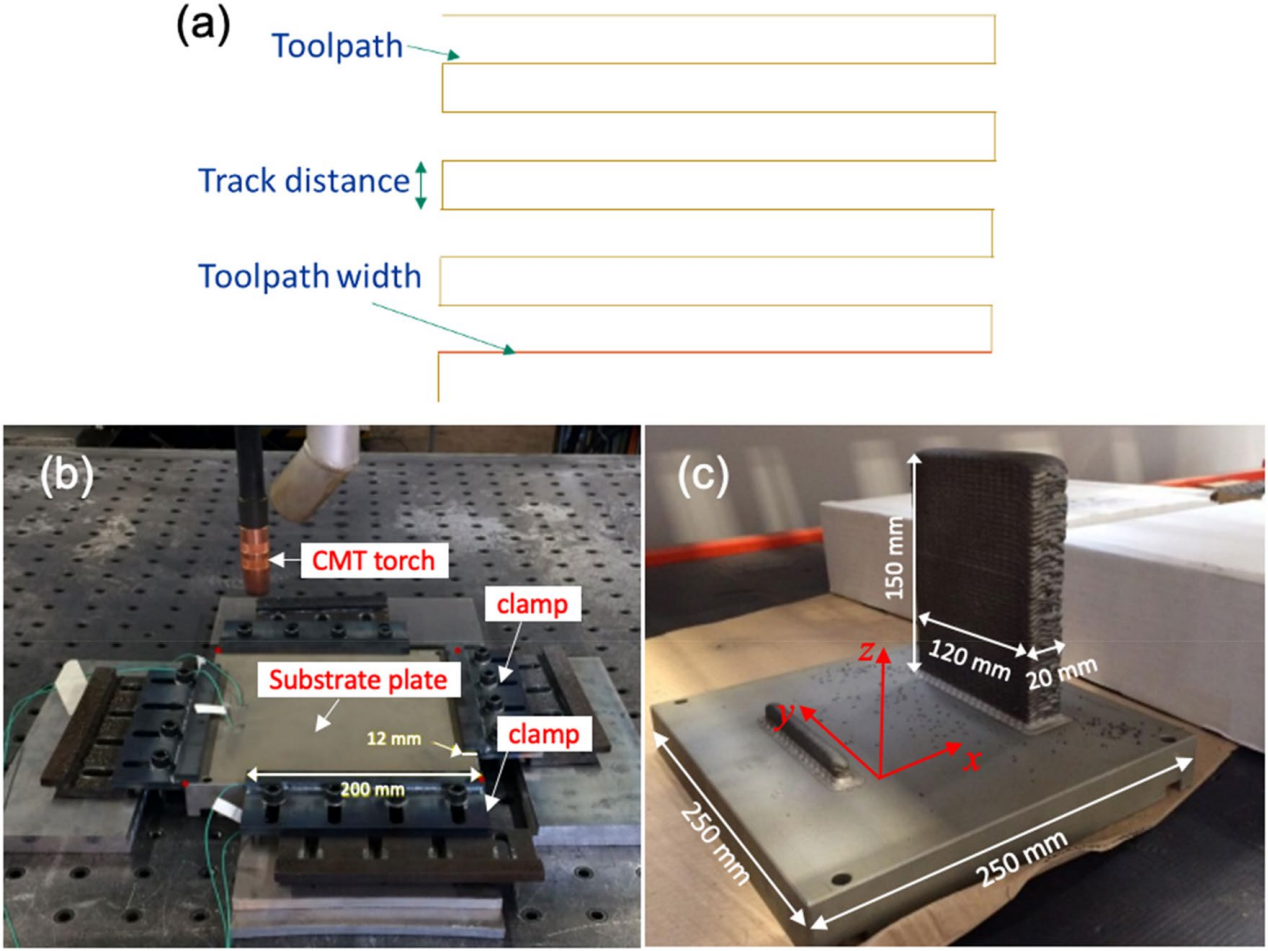
(**a**) Deposition strategy used to manufacture wall, (**b**) printing setup and (**c**) build from manufacturing process of Inconel 718.

A wall of dimensions 20 mm × 120 mm × 150 mm was printed on a Gefertec Arc603 wire arc additive manufacturing machine using 1.2 mm filler wire Alloy Inconel 718 from VDM. The machine setup consists of a gantry-based CNC and a cold metal transfer (CMT) power source from Fronius. The wall was printed using a meander deposition path shown in Fig. 9a. The average energy density used during this build was 30.32 J/m^3^.

The printing process was carried onto a 250 mm × 250 mm × 34 mm mild steel substrate plate. The substrate plate was at room temperature before the printing process started. The clamping setup and result are shown in Fig. 9b,c. This wall was used to harvest samples investigated in this study.

### Heat treatment, microstructural analysis and mechanical testing

The aging process was conducted on the AF samples under the temperature from 500 to 900 °C at an interval of 50 °C for 16 h with air cooling. The AF samples are subject to a) direct aging (DA), b) homogenization followed by aging (HA), where the homogenization treatment is conducted at 1150 °C for 24 h followed by water quenching. In addition, the wrought IN 718 is also homogenization + aging heat treated (HAw) as comparison. Note the HAw condition in our study is not considered as wrought standard.

The AF and wrought specimens with/without heat treatment for mechanical tests were electrical discharge machined (EDMed) along the horizontal directions (*x–y* plane perpendicular to the build direction) in the area at least 10 mm away from the top and substrate. They were prepared following the ASTM E8 standard, with a total length of the sample of 50 mm and the gauge length of 15 mm. The width within the gauge length and that of the grip area were 3 mm and 11 mm, respectively. The thickness of the sample was 2 mm. Tensile tests were conducted under the Instron E3000 machine with a displacement rate of 0.0167 mm/s at the crosshead, equipped with induction heater for elevated temperature condition. Pyrometers and thermocouples were employed for accurate temperature control. The tensile test was conducted in laboratory air condition.

The microstructures of samples were examined by optical microscopy (OM), scanning electron microscopy (SEM) and electron backscatter diffraction (EBSD). The samples for OM/SEM/EBSD analysis were prepared through mounting, grinding, and vibration polish or electropolish. The electropolish were conducted with a solution of 12.5 vol% sulfuric acid and 87.5 vol% methanol at a potential of 25 V at room temperature. The etching was conducted electrolytically in a 10 vol% saturated oxalic acid solution at 12 V for a couple seconds. Samples for transmission electron microscope (TEM) analysis were prepared using a twin-jet polisher with a solution of 10 parts perchloric acid, 45 parts methanol, and 45 parts glacial acetic acid by volume, at 228 K and a potential of 15 V.

## Conclusion

The IN718 wall is successfully fabricated by wire-arc DED. Both direct aging from 600 to 900 °C and homogenization heat treatment followed by aging to dissolve the Laves and other precipitates are applied to the AF 718. A wrought 718 is also investigated as comparison. Their microstructures and mechanical properties at the room and elevated temperatures are investigated. The main conclusions are drawn as follows:

1. The as-fabricated microstructures are featured by large columnar grains with prominent dendrites. Strong texture is developed along the build direction. Two different Laves phases of distinct size, chemical composition, and lattice structures, along with Nb-rich carbides are identified in the AF condition.
2. Direct aging does not remove the inter-dendritic Laves phase and large columnar grain. However, the DA at 700 °C has reached a room-temperature YS_0.2_ of 1026.0 MPa, UTS of 1263.1 MPa and a ductility of 18.6%. These are among the highest values reported for wire-arc DED 718 and comparable to the wrought standard.
3. Direct aging below 750 °C also leads to higher room temperature YS_0.2_ and UTS than the HA conditions without compromising the ductility.
4. The tensile loading conducted at an elevated temperature below 750 °C reveals that the UTS of the DA condition is significantly higher than the aged samples with different pre-conditions, i.e., HA and HAw.
5. The DA condition also demonstrates remarkable ductility when deformed at elevated temperature, compared to those with prior homogenization treatment.
6. The highly non-uniform microstructures featured by dendritic-core ultrafine (nanoscale) γ″ precipitation and inter-dendritic micron-sized Laves/carbides in the direct aged samples are highly likely to be responsible for the observed superior mechanical properties.

This study paves the way to optimize the thermomechanical properties of wire-arc DED 718 and other relevant alloys though tailored microstructures by optimizing the printing parameters and post-processing methods.

### Supplementary Information

Supplementary Figures.

## Data Availability

The datasets used and/or analysed during the current study available from the corresponding author on reasonable request.
