# Supplementary material for: Unusually high room and elevated-temperature tensile properties observed in direct aged wire-arc directed energy deposited Inconel 718
Source: Sci Rep. 2023 Nov 6;13:19235. doi: 10.1038/s41598-023-46674-z (PMC10628216; doi:10.1038/s41598-023-46674-z)
Supplement: Supplementary file 1 — Supplementary Figures. [file 41598_2023_46674_MOESM1_ESM.docx]

**Unusually High Room and Elevated-Temperature Tensile Properties Observed in Direct Aged Wire-Arc Directed Energy Deposited Inconel 718**

Jie Song ^a^, Xavier A. Jimenez ^b^, Carissa Russell ^c^, Albert C. To ^b^, Yao Fu ^a,d,*^

1. Department of Aerospace and Ocean Engineering, Virginia Tech
2. Department of Mechanical and Materials Engineering, University of Pittsburgh
3. Materials Sciences LLC
4. Department of Materials Science and Engineering, Virginia Tech


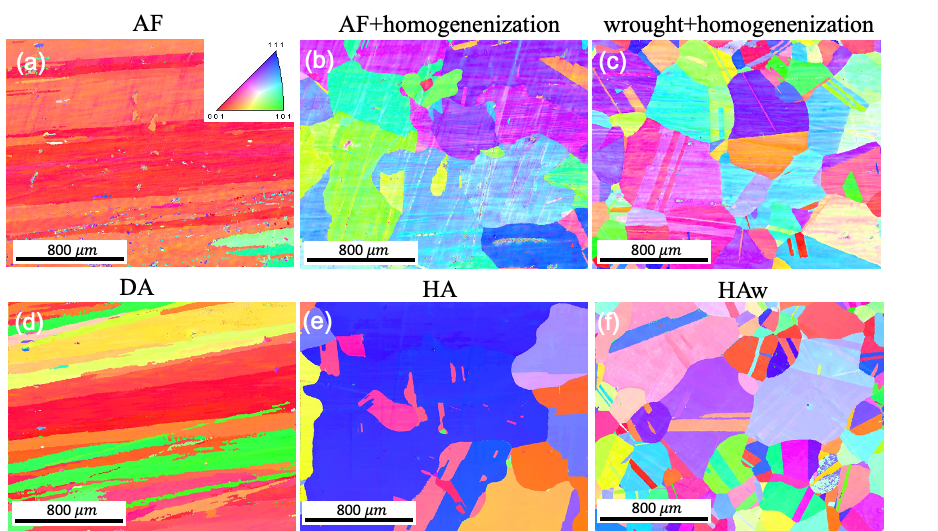


Figure S1 EBSD-IPF images of the (a) AF, (b) AF+homogenenization, (c) wrought+homogenenization, (d) DA, (e) HA, and (f) HAw at the 700 °C


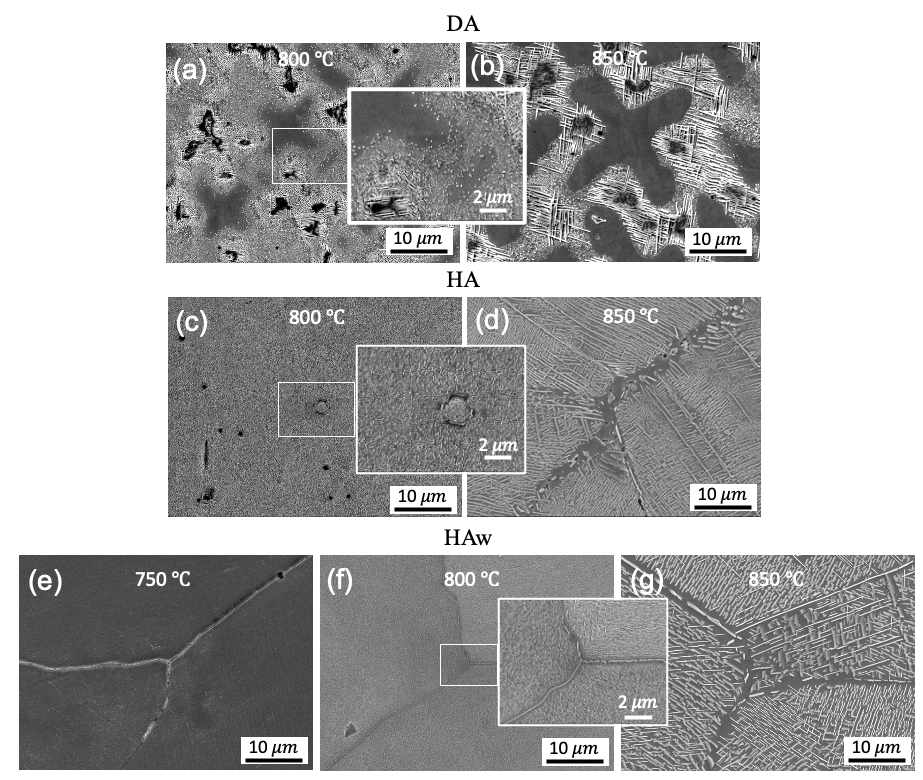


*Figure S2. SEM images of the DA 718 at the (a) 800 °C, (b) 850 °C aging; SEM images of the HA 718 at the (c) 800 °C, (d) 850 °C aging; and SEM images of the HAw 718 at the (e) 750 °C, (f) 800 °C, and (g) 850 °C aging; insert is the magnified image of the white rectangular domain. Aging time is 16 hours (16H) unless marked differently in the figure.*
